# Structural Insight of a Trimodular Halophilic Cellulase with a Family 46 Carbohydrate-Binding Module

**DOI:** 10.1371/journal.pone.0142107

**Published:** 2015-11-12

**Authors:** Huaidong Zhang, Guimin Zhang, Chaoxiang Yao, Muhammad Junaid, Zhenghui Lu, Houjin Zhang, Yanhe Ma

**Affiliations:** 1 State Key Laboratory of Microbial Resources, Institute of Microbiology, Chinese Academy of Sciences, Beijing, China; 2 Department of Biotechnology, College of Life Science and Technology, Huazhong University of Science and Technology, Wuhan, Hubei, China; 3 Key Laboratory of Molecular Biophysics, Ministry of Education, Wuhan, Hubei, China; 4 Hubei Collaborative Innovation Center for Green Transformation of Bio-Resources, College of Life Science, Hubei University, Wuhan, China; Universitetet i Bergen, NORWAY

## Abstract

Cellulases are the key enzymes used in the biofuel industry. A typical cellulase contains a catalytic domain connected to a carbohydrate-binding module (CBM) through a flexible linker. Here we report the structure of an atypical trimodular cellulase which harbors a catalytic domain, a CBM46 domain and a rigid CBM_X domain between them. The catalytic domain shows the features of GH5 family, while the CBM46 domain has a sandwich-like structure. The catalytic domain and the CBM46 domain form an extended substrate binding cleft, within which several tryptophan residues are well exposed. Mutagenesis assays indicate that these residues are essential for the enzymatic activities. Gel affinity electrophoresis shows that these tryptophan residues are involved in the polysaccharide substrate binding. Also, electrostatic potential analysis indicates that almost the entire solvent accessible surface of CelB is negatively charged, which is consistent with the halophilic nature of this enzyme.

## Introduction

With the increasing energy cost, dwindling oil fuel reserve and ever-worsening problem of pollution, the search for a replacement for the fossil fuels has become an urgent task. Due to the abundant lignocellulosic substance in the biosphere, the production of biofuel with cellulose has emerged as a promising solution[1, 2]. The building blocks of cellulose are glucose molecules, which is a good raw material for fermentation. However, the cellulose consists of straight chain of glucose polymers. These polymers form rod-like structures which are strengthened by the multiple hydrogen-bonds between or within the polymers. In the cell wall of plant, microfibers of cellulose crosslink with hemicellulose and lignin to form a resilient biopolymer matrix. The crystalline nature of this matrix makes it difficult to degrade cellulose into glucose units[3]. Various physical and chemical methods have been developed to release the sugar molecules from biomass, however, the bottleneck is the cost-effective and efficient enzymes for industrial-scale conversion of lignocellulose to fermentable sugars[4]. The natural degradation of cellulose is the result of a group of glycoside hydrolases (GHs) working in synergy. Although the exact mechanism of cellulose hydrolysis is difficult to establish due to the complexity of the substrate, several key steps are involved. Firstly, the cellulose fibers are cleaved into fragments by endoglucanases. The shortened cellulose is clipped by the cellobiohydrolases from the end. The resulting cellobiose is then hydrolyzed into glucose by beta-glucosidases[5]. In the industrial setting, similar group of enzymes are used together to digest cellulose. However, the enzymes are put into harsh environments such as high temperature, high salt and acidic/basic conditions. Significant engineering efforts have been made to improve the properties of natural enzymes in order to meet such requirements in the industrial applications[6]. Alternatively, a good source of these industrial enzymes can be found in the microorganisms living in extreme conditions[7]. For example, a thermo-stable cellulase CelDR with optimum temperature of 50 degree centigrade was found in a strain isolated from a hot spring[8]. In addition, the function-based mining of metagenomes from the soil samples in a cold desert resulted in the discovery of an acidic and cold-active cellulase[9]. In our previous study, a halophilic cellulase was identified from the genome library of *Bacillus* sp. BG-CS10, an alkaliphilic and halophilic *Bacillus* strain from a Tibetan salt lake. This cellulase, CelB, is thermo-stable, halophilic and pH-tolerant. CelB can utilize soluble cellulose derivatives, such as carboxylmethyl cellulose and konjac glucomannan, while it can not hydrolyze insoluble cellulose derivatives, such as microcrystalline and cellulose CM-52. Also it has only endoglucanase activity with no exoglucnasase activity detected[10]. Interestingly, its activities are increased 10 fold after addition of 2.5 M NaCl or 3M KCl, which is very rare among cellulases [10].

The two main groups of cellulases are exoglucanases and endoglucanases. A typical exoglucanase has a globular structure with an active site tunnel going across it. The tunnel is surrounded by several anti-parallel beta-sheets. Within the tunnel lies the conserved Trp patches which serve as the anchoring point for the substrates. To accommodate the substrate in the tightly packed tunnel, several loops are located around the tunnel and provide flexibility for the region[11, 12]. In contrast, the structure of a typical endoglucanase has a shallow cleft instead of a tunnel at the active site. Compared to the tunnel found in the exoglucanases, the active site cleft of a endoglucanase provides an easy access for the cellulosic fibers which is still in a tightly packed state[13]. Also in the tunnel are the catalytic residues including two acidic residues such as aspartic acid or glutamic acid[14]. One carboxylate residue serves as the nucleophile and the other serves as the catalytic base. Although the carboxylate base/nucleophile is the classical combination which is found at the active site of most GHs, some diversity can be found in the active site. In some cases, no nucleophile is found at the active site. Instead, the carbonyl oxygen of the substrate acts as the nucleophile and forms oxazoline intermediate[15]. In GH-6 cellulases, a proton transfer network replaces the role of carboxylate base[16]. In the glycosidases from clan GH-E, the nucleophile is a Tyr instead of Glu. In some cases, the catalytic residues consist of an Asp-His dyad. In a beta-N-acetylglucosaminidase from *Bacillus subtilis*, the normal dual-acidic catalytic residues are replaced with an aspartic acid residue and a histidine residue. In such configuration, the histidine functions as a proton donor while aspartic acid is a nucleophile[17]. Also, some cofactors such as phosphate and NAD can act as exogenous base or nucleophile[18, 19].

The catalytic domains of GHs can perform the hydrolysis of glycosidic bond by itself, presumably due to the presence of aromatic residues which can bind to the glucosic moiety through CH-pi hydrogen bonds. However, many GHs possess carbohydrate-binding modules (CBMs) that connect to the catalytic domains through linker sequences. The CBMs facilitate the GHs to bind to the substrate, therefore, increases the catalytic efficiency. The CBMs are classified into 71 families based on the sequence similarities[20]. High-resolution structures are available for many CBM families. Based on the structural similarity, CBMs can be divided into several fold families[21]. The most common fold is the β-sandwich fold which comprises two β-strands on top of each other. The other fold families include β-trefoil fold, cellulose binding fold and hevein fold families. According to the architecture of the binding sites, the CBMs are classified into three types. The type A CBMs, with a flat binding surface, prefer to bind highly crystalline cellulose and chitin. The type B CBMs have a binding grooves or clefts which are the docking sites for extended carbohydrate polymer chains. In contrast, the type C CBMs bind optimally to the mono-, di- or tri-saccharides[21].

To understand the spacial arrangement and the functions of individual domains in CelB, we have solved the structure of the trimodular cellulase CelB. The structural analysis indicates that the three domains (catalytic, CBM_X, CBM46) form a tightly-packed L-shape structure. The catalytic domain and the CBM46 are located next to each other and an extended substrate-binding cleft is found between them. Inside of the cleft are several tryptophan residues which are proven to be important for substrate binding. Also, almost the entirely surface of CelB is negatively charged, which is a signature of halophilic proteins.

## Materials and Methods

### Construction of Expression Vectors

The coding sequence for mature CelB was amplified from the genome of *Bacillus* sp. BG-CS10, ligated into pET-28a(+) vector and sequenced for correction [10]. The pET-28a(+) vector with the *celB* gene was transformed into *Escherichia coli* strain BL21(DE3) Rosetta for protein expression. The single-point mutations were introduced by PCR-based site-directed mutagenesis method. The primers used are listed in S1 Table. Eighteen mutants were constructed including W37A, H103Q, H104Q, W107A, E149Q, W156A, H224Q, W229A, E271Q, W310A, H349A, H364A, H366A, W425A, H426A, W476A, Y484A, Y490A (S1 Table). The resulting plasmids were amplified in DH5 α. The mutations were confirmed by DNA sequencing.

### Expression and Purification of CelB

The bacteria were grown overnight at 37°C in Luria Bertani (LB) broth with kanamycin (50 μg/mL) and chloramphenicol (100 μg/mL), and inoculated into LB broth with kanamycin (50 μg/mL) and chloramphenicol (100 μg/mL). The culture was incubated at 37°C, 200 r·min^-1^ in shaker flasks. When the optical density at 600 nm (OD_600_) reached 0.6, IPTG was added into the medium up to 0.1mM. The protein CelB expression was induced at 16°C, 200 r·min^-1^ for 16h. The culture was collected by centrifugation at 6,000 g at 4°C for 10 min after induction and the pellet was resuspended in 20 mM Tris-HCl, pH 8.3, 500 mM KCl. The cells were lysed by ultrasonication. And the cell debris and the supernatant were separated by centrifugation at 4°C, 13,800 g for 30 min.

The supernatant was applied to Ni-NTA Resins (GE Healthcare) pre-equilibrated with 20 mM Tris-HCl, pH 8.3, 500 mM KCl and then washed with 20 mM Tris, pH 8.3, 500 mM KCl, 30 mM imidazole. After elution with 20 mM Tris-HCl, pH 8.3, 500 mM KCl, 200 mM imidazole, the His-tag was removed with thrombin (1 unit/mg, Sigma). The protein was dialyzed with 20 mM Tris-HCl, pH 8.3, 200 mM KCl and loaded to a Superdex 75 column (16/60, GE Healthcare) equilibrated with 20mM Tris-HCl, pH 8.3, 100 mM KCl [22]. Then 2 M ammonium sulfate was added into the protein solution. The mixture was loaded onto a hydrophobic chromatography (Phenyl Sepharose6 Fast Flow, GE Healthcare) and eluted with 50 mM Tris-HCl, pH 7.0. The purity and molecular weight of the protein sample were analyzed by SDS-PAGE. The protein was concentrated by 10K, Amicon^®^ Ultra-4 Centrifugal Filter Units, and the protein concentration was estimated spectrometerically with OD_280_ [23]. The selenomethionine-labeled CelB was expressed as described in the literature and purified as native protein[24]. In brief, 2 ml culture was used to inoculate 2L M9 medium supplemented with 50 μg/mL kanamycin, 2 mM MgSO_4_, 0.1 mM CaCl_2_ and 5 g/L dextrose. The culture was grown at 37°C until OD_600_ reached 0.8. Then lysine, phenylalanine and threonine were added at 100 mg/L, leucine, isoleucine and valine were added at 50 mg/L, then L-selenomethionine was added at 40 mg/L. After incubation for 15 min, 0.1 mM IPTG was added to induce the protein expression and the incubation was continued for another 12 hours at 16°C.

### Crystallization and Data Collection

Crystals of native CelB were grown by the sitting-drop vapor diffusion method. Briefly, 1 μL of protein (10 mg/mL) was mixed with the precipitant solution containing 2.0 M ammonium sulfate, 5% (v/v) 2-propanol. The crystals of CelB appeared after 7 days incubation at 289.15 K and reached their maximum size 10 days later. Selenomethionine-labeled CelB crystals were grown in the same manner, and the precipitant solution was optimized by adding 0.1 M potassium sodium tartrate tetrahydrate. A cryo-protectant solution was made by supplementing precipitant solution with 25% (v/v) glycerol (Sigma). The crystals were immersed in the cryoprotectant briefly before frozen in liquid nitrogen.

Diffraction data was collected at 100K from selenomethionine-labeled CelB crystals at the selenium absorption edge with a Quantum 315 CCD detector (ADSC) at a wavelength of 0.979197 Å on beam-line BL17U, Shanghai Synchrotron Radiation Facility (SSRF), Shanghai, China. The data set was indexed and integrated with XDS package [25] and scaled with Aimless [26]. Initial phases were obtained by single-wavelength anomalous diffraction (SAD) using the anomalous scattering from the selenomethionine incorporated at the methionine sites. All 13 selenium sites in the asymmetric unit were located with PHENIX package and the phase was refined to a figure of merit of 0.284[27]. The phase was further improved with DM program in the CCP4 package (figure of merit = 0.782)[26]. The resulting electron density map was of very good quality and side-chains of 80% residues could be automatically built with PHENIX[27]. The manual building was done with Coot[28] and the structure was refined with PHENIX after each building cycle[29]. Portion of the data (5%) was set aside to calculate free R factor, which was used to monitor the bias throughout the model building process[30]. The stereochemistry of the model was validated at the late stage of manual building with MolProbity[31]. Native data set was collected at 100 K with a Rigaku R-AXIS IV++ detector at Public Technology Service Platform, Wuhan Institute of Biotechnology, Wuhan 430074, China. The native structure was solved by molecular replacement with Phaser, using selenomethionine-labeled CelB structure as the search model[32]. Data parameters and refinement statistics are summarized in Table 1.

**Table 1:** Data collection and refinement statistics.

	Native CelB	SeMet CelB
**Data collection**		
Space group	I 4 2 2	I 4 2 2
Cell dimensions		
a, b, c (Å)	120.14, 120.14, 205.33	120.99, 120.99, 205.00
α, β, γ (°)	90.00, 90.00, 90.00	90.00, 90.00, 90.00
Resolution (Å)	65.45–2.35(2.43–2.35)	85.55–2.34(2.43–2.34)
Wavelengths(Å)	1.5418	0.9791
Rmerge^1^	0.10(0.66)	0.09(0.87)
Mean I/σ(I)	19.40(2.50)	11.60(2.02)
Completeness (%)	98.20 (95.50)	99.70(100.00)
Redundancy	7.70(5.60)	6.30(6.50)
Wilson B-factors (Å^2^)	30.70	43.88
Anomalous completeness(%)		99.20(100.00)
Anomalous Redundancy		3.30(3.30)
FOM/DM FOM^2^		0.28/0.78
**Refinement**		
Resolution (Å)	65.45–2.35(2.43–2.35)	43.96–2.34(2.43–2.34)
No. of total reflections	262046(18271)	103130(6616)
No. of unique reflections	30948(2956)	32069(940)
R_work_ / R_free_ ^3^	0.18/0.23	0.19/0.23
Number of atoms		
Protein	4312	4312
Water	461	196
Average B-factor (Å^2^)		
Protein	31.00	48.40
Water	36.50	51.40
R.m.s deviations		
Bond lengths (Å)	0.007	0.006
Bond angles (°)	1.03	0.99
Ramachandran plot^4^		
Allowed region (%)	3.60	4.35
Favored region (%)	96.40	95.65
Outliers (%)	0.00	0.00
PDB ID	5E0C	5E09

^1.^ Data in the parenthesis was calculated based on the highest resolution shell.

^2.^ Mean figure of merit with or without density modification.

^3.^ R-factor = (Σhkl||Fo|-|Fc||)/Σhkl|Fo| where Fo and Fc are the observed and calculated structure factors respectively. R_free_ was calculated with a randomly-selected 5% subset which was excluded from the refinement process.

^4.^ Statistics of Ramachandran plot were calculated with MolProbity.

### Enzymatic Activity Assay and Gel Affinity Electrophoresis

The enzymatic activity was evaluated by measuring the reducing sugars released from substrate carboxymethylcellulose (CMC). Purified enzymes (native or mutant) were added to a reaction mixture containing 1% CMC, 2.5M NaCl, phosphate buffer, pH 5.0. The mixture was incubated at 55°C for 30 min. And the amount of reducing sugars was measured by the dinitrosalicylic acid reagent (DNS) method.

Affinity gel electrophoresis was used to investigate the binding affinity between CelB mutants and soluble polysaccharides. Proteins were resolved on nondenaturating polyacrylamide gels 10% (w/v) containing 0.3 mg/ml of hydroxyethyl cellulose (HEC). Electrophoresis was carried out for 5h at room temperature.

### Molecular Dynamics Simulation

Molecular dynamics simulation of CelB was carried out in high salt concentration using *pmemd*.*cuda* [33] module of Amber14 [34]. The protein was solvated in a rectangular box filled with TIP3P water molecules using tleap module of Amber14 [35]. A buffer distance of 12 Å was set between the protein edge and the box boundary in all directions. NaCl salt molecules were added to reach 1M concentration. Amber ff14SB force field was used to generate coordinate and topology files for the protein [36]. In order to remove bad contacts between solvent and protein, energy minimization was carried out in two steps. Firstly, the system was minimized keeping the protein fixed with harmonic constraint of a strength of 500 kcal·mol^-1^·Å^-2^. Secondly, the whole system was minimized without any constraint. The above each step was performed with the steepest descent minimization of 1000 steps followed by a conjugate gradient minimization of 1000 steps. The system was then heated to 300K in 2000 steps. Finally the system was simulated for 50 ns and the trajectory was saved after each 20 ps. The SHAKE algorithm was used for the covalent bonds involving hydrogen [37]. The Particle Mesh Ewald (PME) method was adopted to treat the long-range electrostatic interactions [38].

## Results

### The Overall Structure of CelB

The CelB was expressed in *E*. *coli* BL21(DE3) Rosetta, purified and subjected to crystallization screening. The crystals of CelB appeared after 7 days incubation at 289.15 K in the sitting-drop crystallization plates and reached their maximum size 10 days later. The CelB crystal belongs to the body-centered tetragonal system (I 4 2 2), with unit-cell parameters a = b = 120.14 Å, c = 205.33 Å. The data-collection and processing statistics are presented in Table 1.

**Fig 1:**
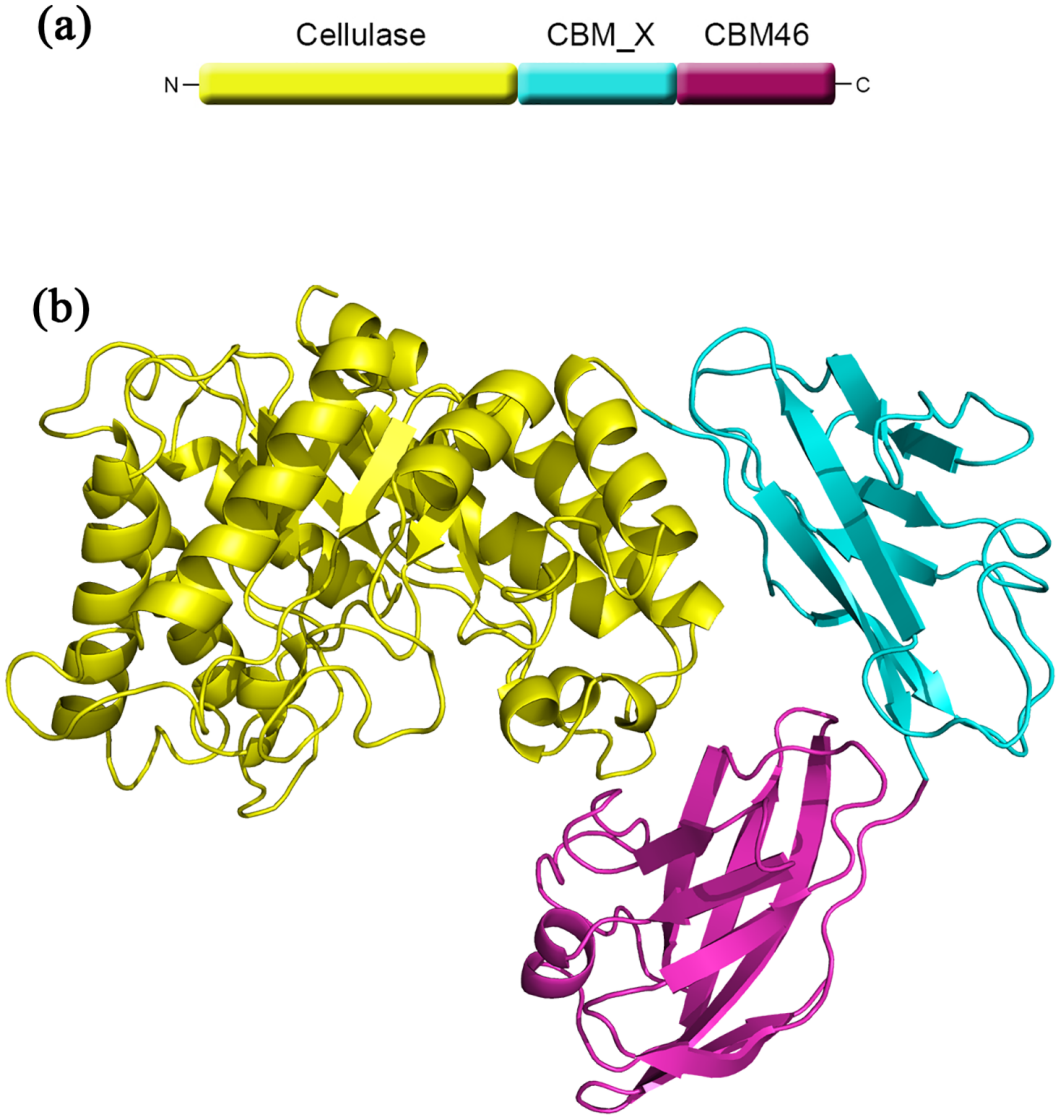
Overall structure of CelB. (a) The domain arrangement in the primary structure of CelB. The cellulase catalytic domain, colored in yellow, is located at the N-terminus, the CBM46 domain, colored in red, is located at the C-terminus and the CBM_X domain is located in the middle portion of the protein. (b) The cartoon representation of CelB overall structure. The catalytic domain is in yellow, the CBM46 domain is in red and the CBM_X domain is in cyan. The catalytic domain and the CBM46 domain form an extended substrate-binding cleft. CBM_X domain serves as the rigid connection between the first and the third domains.

The overall structure of CelB consists of a typical (β/α)_8_ TIM barrel catalytic domain, a CBM_X domain and a CBM46 domain (Fig 1). The substrate-binding sites are composed of a deep cleft across the catalytic domain. The cleft is formed by several loops and small α helices on top of the barrel. Deep inside of the cleft are the catalytic residues including Glu149 and Glu271. Also in the active sites are several well-exposed aromatic residues serving as the anchoring points for the polysaccharide molecules. The CBM_X domain is located right next to the catalytic domain. The connection between these two domains is established through hydrogen bonds between Arg240/Glu344, Lys219/Glu344, Gln285/Asn346, and the N-H/pi interaction between Arg339/His366. Unlike most CelB with CBM attached, there is no linker present between CBM_X domain and catalytic domain. There is a loop (Gly338-Leu347) at the N-terminus of CBM_X domain. However, this loop is tightly tethered to the CBM_X domain as well as the catalytic domain. Therefore, it does not function as a conventional loop. Also tightly connected is the CBM46 in the molecule. There is no linker between the CBM_X and CBM46 domains either. The CBM46 domain forms hydrogen bonds with both the CBM_X domain and catalytic domain. As a result, instead of forming an extended chain, these three domains form a tightly packed L-shaped structure. More importantly, the aromatic residues on the CBM46 (Phe470, Trp476) are close to the catalytic cleft and form an extended binding site for the substrate.

### The Catalytic Center and Substrate-binding Cleft

**Fig 2:**
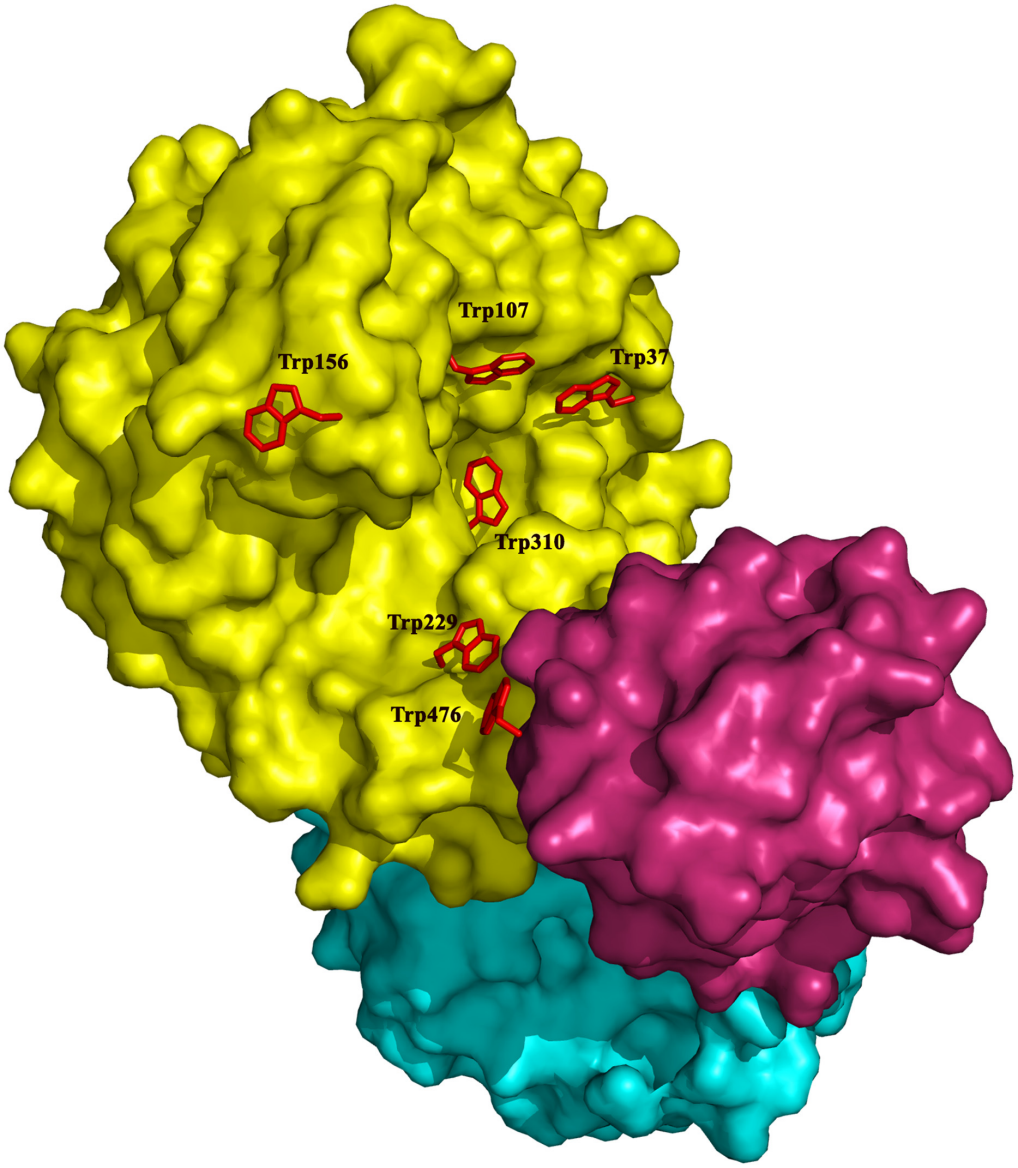
Surface representation of CelB. The catalytic domain is colored in yellow, the CBM_X domain is colored in cyan and the CBM46 domain is colored in red. The tryptophan residues around the substrate binding cleft are shown in sticks and colored in red. The rest of molecule is shown as surface.

The catalytic domain of CelB (residues 1–340) adopts a typical TIM barrel fold that is found in many GHs of GH5 family. In addition to the canonical (β/α)_8_ fold, some additional structural elements are also found in the catalytic domain, such as two 3/10 helices (residues 275–278 and 281–283) as well as an extended loop (residues 145-159) between beta strand 4 and alpha helix 6 (S1 Fig). These elements are located at the entrance of the TIM barrel and significantly deepen the substrate-binding cleft. The Dali secondary structure comparison performed with the catalytic domain shows that it is homologous to various endoglucanases[39]. The closest structural homolog is the endoglucanase D (engD) from *Clostridium cellulovorans*, with a RMSD of 2.1 Å. Several tryptophan residues (Trp37, Trp107, Trp156, Trp229, Trp310 and Trp476) are located on the surface of the substrate-binding cleft (Fig 2). These aromatic residues presumably serve as the attaching points for the polysaccharide substrate. Except for Trp107 and Trp476, these aromatic residues occupy similar positions as those in the active site of engD, which implies similar substrate binding patterns of these two cellulases. Actually, Trp107 is conserved in other GH5s [40]. In engD, Trp162 and Tyr232 form a clamp that encloses the substrate-binding cleft [41]. However, in CelB, due to the shift of a long loop, such clamp is not present, which results in a more open active site. The superposition of CelB and engD structures indicates that the Trp37 and Trp310 of CelB overlap with the corresponding residues in engD. These two tryptophan residues form the -3 and -2 sub-sites for the substrate. The His103 and Asn148 of CelB also overlap with their counterparts in engD, which form the -1 sub-site.

**Fig 3:**
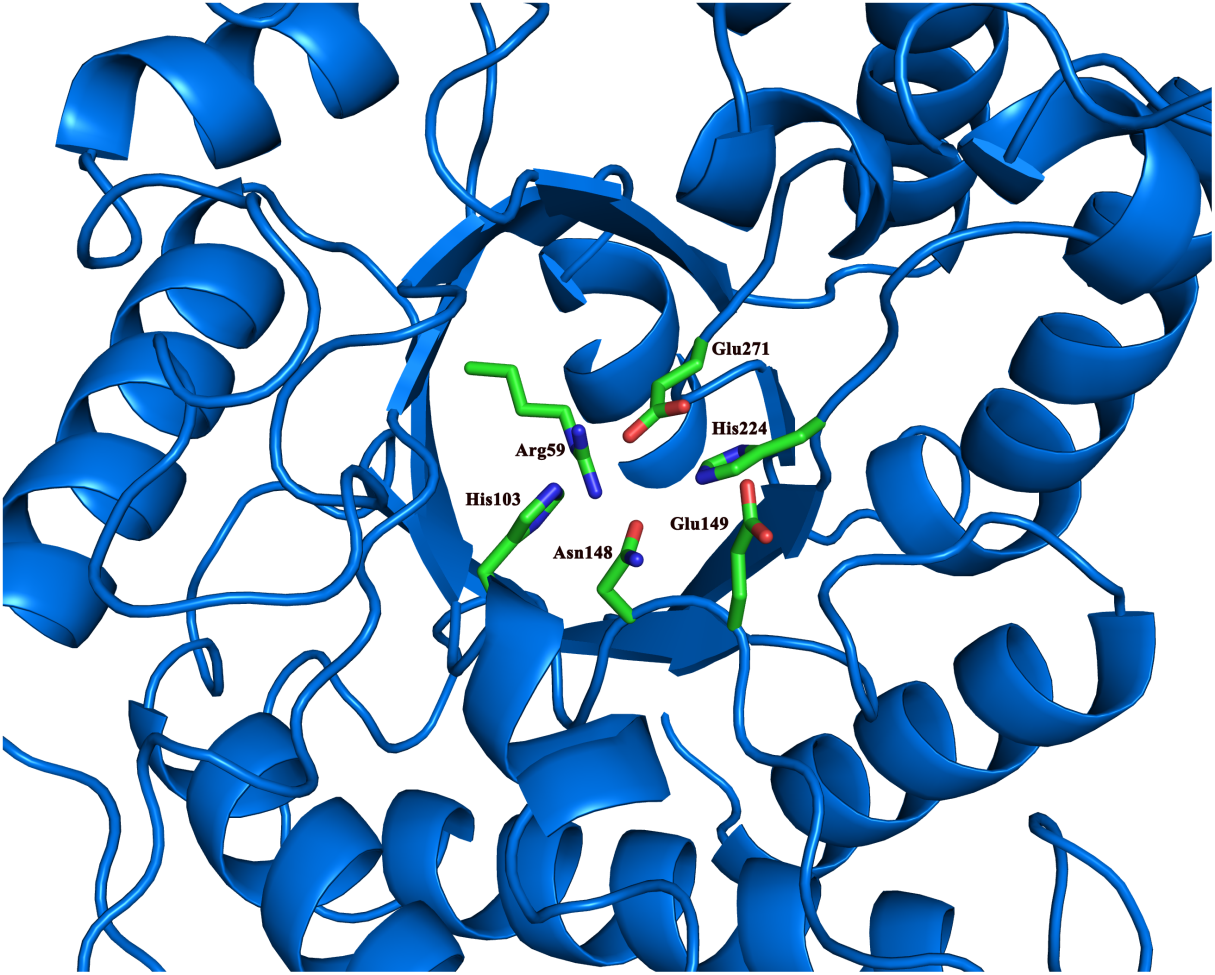
The conserved residues in the catalytic domain. The conserved residues around the two catalytic Glu residues are shown in sticks and colored by elements. The rest of the catalytic domain is shown in ribbons and colored in blue.

At the bottom of the active site are the catalytic Glu149 and Glu271 that are present in most of GHs. In the vicinity of these two catalytic residues is the His224 that is conserved in several GH5 family GHs[13, 41] (Fig 3). Other conserved residues close to the catalytic residues are Arg59, His103 and Asn148. The His103 and Asn148 residues are likely to function as binding sites for the substrate through H-bonds. The Arg59 residue forms several H-bonds with the surrounding residues, including Asn22, Glu145, Asn148, and Glu271. It is possible that Arg59 serves as a pillar to maintain the active site structure.

### The Carbohydrate Binding Modules

**Fig 4:**
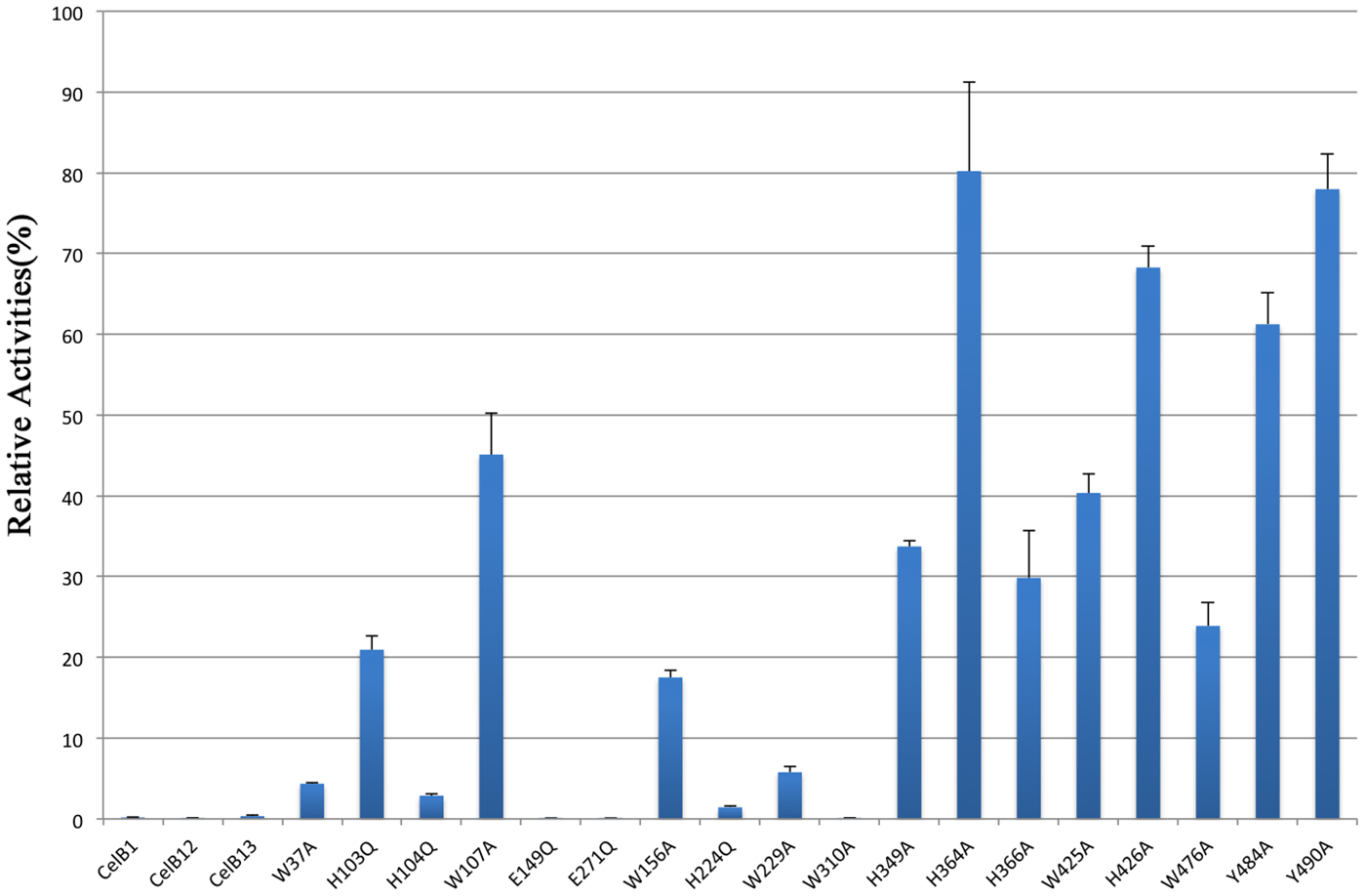
Histogram representation of relative activities of CelB mutants. The activities of mutants were represented as the percentage of native enzyme activity. The data is the average of at least three individual experiments. The error bars show the standard deviation (S.D.) of different repeats of the same assay. CelB1 designates the catalytic domain alone. The CelB12 designates the truncated protein without the CBM46 domain. The CelB13 designates the fusion protein containing the catalytic domain and the CBM46 domain.

A distinctive structural feature of CelB is the CBM_X domain and the CBM46 domain at the C-terminus (Fig 1). The CBMs usually play supplementary roles in the cellulase functions. The absence of CBM only has modest effect on the enzyme activities. However, the truncation experiments indicate that these CBMs are essential for CelB’s function (Fig 4). The deletion of either CBM abolishes the enzyme activities. Sequence analysis shows that the first putative CBM, CBM_X, belongs to an uncharacterized CBM family, while the second putative CBM belongs to CBM family 46 [20]. Unlike the typical CBM, the CBM_X does not form a beta-sheet sandwich. Instead, it has a continuous beta-sheet and forms a barrel-like structure. The Dali secondary structure comparison shows that its closest structural homolog is the neural cell adhesion molecule 2 which has an Ig-like fold[39].

In contrast, the second CBM domain, CBM46, possesses multiple tryptophan, tyrosine and phenylalanine residues on the surface, which is a common feature for CBMs. The mutagenesis experiments indicate that the removal of the aromatic side-chains diminishes the enzymatic activities (Fig 4). Given the locations of these residues, it suggests that these aromatic residues are the docking sites for the carbohydrate substrates. The structure of CBM46 consists of a large anti-parallel beta-sheet rolled into a barrel. It also possesses one alpha helix and several long loops connecting the beta-strands. The structure is stabilized by a network of hydrogen bonds. Also stacking interaction is found between Phe453 and Tyr490, which contributes to the stability of the structure. Although the Blast search does not assign any specific function for this domain, in the carbohydrate-active enzymes database (CAZY), this region of the CelB is classified as the CBM46 group member [20]. As indicated by CAZY, the CBM46 domain is often found in multiple-domain cellulases, which matches the characteristics of CelB. It noteworthy that neither CBM_X domain nor CBM46 has the DxDxDG calcium-binding motif and no calcium is found in the structure of CelB. This is also different from many classic CBMs among which calcium plays an important role in structural stability and substrate recognition[42–44].

### Structural Basis for the Salt Resistance of CelB

**Fig 5:**
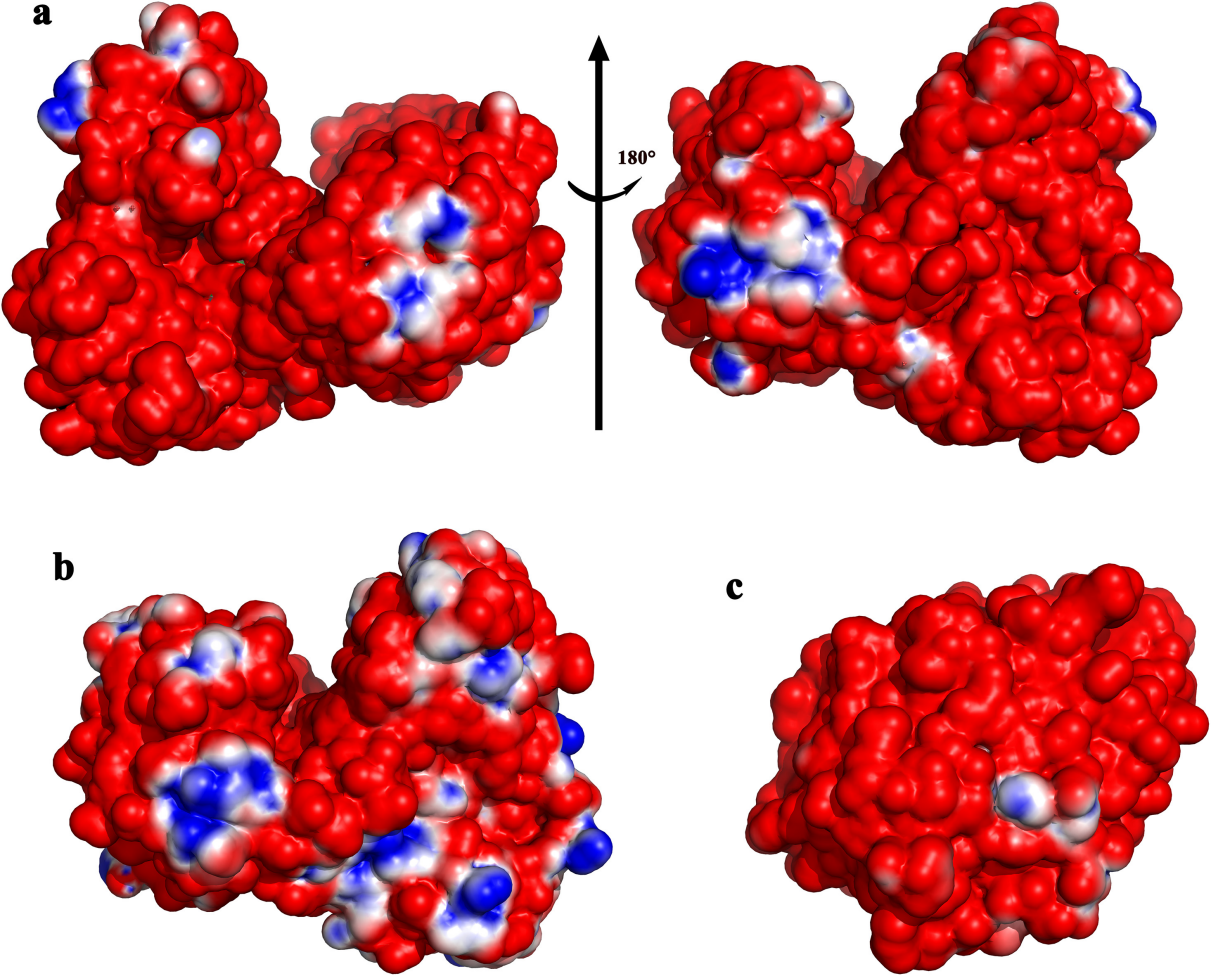
Surface electrostatic potential of CelB and other haloduric cellulases. The electrostatic potential between -1 kT/e and 1 kT/e is shown as a colored gradient from red (acidic) to blue (basic). (a) The left view is rotated 180 degrees from the right view. Almost all the surface of CelB has negative electrostatic potential. Only small patches of positive electrostatic potential can be found. (b) The surface electrostatic potential of Cel5B from *Bacillus halodurans*. (c) The surface electrostatic potential of LqCel7B from *Limnoria quadripunctata*.

Electrostatic potential analysis indicates that almost the entire solvent accessible surface of CelB is negatively charged (Fig 5). Some small positive patches are found at two groups of arginine residues including Arg41/Arg44/Arg83, Arg536/Arg537 and a group of charged residues including Lys21/His208. The structural analysis of a close homolog, BhCel5B from *Bacillus Haloduran* C-125, shows that its surface is also negatively charged, indicating it is a common feature for this group of cellulase extracted from high-salt environment[45]. The predominantly negative charge is attributed to the large amount of acidic residues present on the protein surface. Sequence analysis shows that the acidic residues (Asp and Glu) account for 16.3% of the total residues of CelB. A direct consequence of exposed acidic residues is the low isoelectric point (pI) of CelB. The predicted pI of CelB is only 4.53 as predicted by ProtParam[46]. This is lower than other GH5 cellulases of the similar size and modular composition. The negatively charged residues on the surface and low pI should be the result of high-concentration of salt in the environment it exists[10]. Actually, the highly acidic surface is the hallmark of many salt-resistant proteins[11]. Like CelB, some of these GHs are not only resistant to salt, but also activated by the increased salt concentrations, which makes them promising candidates for biocatalyst used in extreme conditions [11, 47, 48].

### Site-directed Mutagenesis and Truncation of CelB

Site-directed mutagenesis and truncation were used to investigate the importance of key residues in the catalytic domain as well as two CBM domains. The residues close to the catalytic Glu residues were mutated to Gln instead of Ala, in order to keep the length of the side-chains approximately the same. The mutants were used to carry out enzymatic assays in the same experimental setting as the native enzyme. The relative activities of the mutants were calculated as the percentage of the native enzyme activity (Fig 4). The CelB1 mutant, which only contains the catalytic domain, has no activity. The CelB12 mutant, which is the truncated protein without CBM46 domain, does not have any activity. The fusion protein of catalytic domain and the CBM46 (designated as CelB13) does not have any activity either. Therefore, the tri-modular CelB possess a long active-site cleft that is formed by all three domains. The absence of any domain abolishes the enzyme activity. It is thermodynamically unfavorable to have the bulky hydrophobic tryptophan residues exposed to the solvent. In the catalytic domains of cellulases, such exposed tryptophan residues are often observed, which are proven to serve as the binding sites for the polysaccharide. The tryptophan residues in the catalytic domain were systematically mutated to alanine to investigate the functions of their bulky side chains (W37A, W107A, W156A, and W229A). As expected, the subsequent enzymatic assay indicates that these tryptophan residues are essential to the enzyme activities. The activities of these mutants are significantly reduced, compared to the native enzyme. In particular, the W37A and W229A mutants only have less than 10% of the original activity (Fig 4).

The histidine residues around the catalytic residues are also mutated (H103Q, H104Q, H224Q). In the canonical cellulase catalytic mechanism, the histidine residues are not included. However, a recent study on another GH5 cellulase has shown that the mutations on His residues close to the catalytic residues can deactivate the enzyme[49]. Histidine residues may serve as a part of proton transfer network, or part of the hydrogen bonding network which maintains the stability of the active site. In GH subfamily GH5_36, arginine instead of histidine is conserved at this position. The replacement of arginine with histidine preserves part of the enzyme activity, suggesting arginine and histidine serve similar roles in the proton transfer network[50]. His103 and His104 residues are well exposed in the active site. They may bind to the substrate through hydrogen bonds. They could also shuffle the protons into the surround solvent. However, H224 is partially shielded. Early research regarding hyperthermophilic endo-β-1,4-glucanase has suggested the two glutamic acid residues and the histidine residue right next to it may form a catalytic triad. But the exact role of this histidine in CelB is yet to be determined.

**Fig 6:**
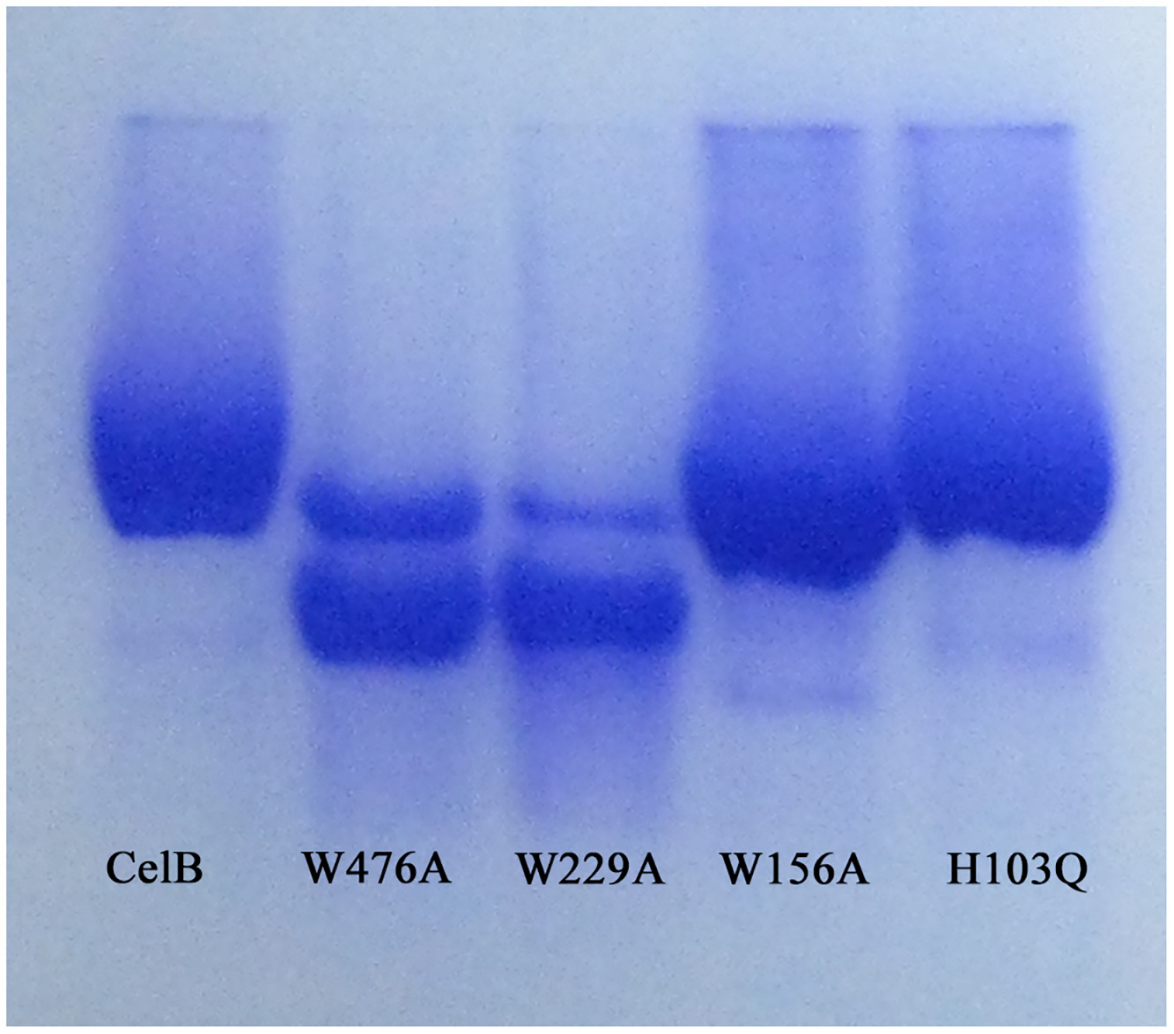
Affinity gel electrophoresis of CelB mutants. The CelB mutants were electrophoresed on a 10% (w/v) nondenaturating polyacrylamide gel containing 0.3 mg/ml of HEC. The native CelB protein was used as the control.

CBM46 domain is a member of CBM family 46. Like the other CBM members, this domain has several aromatic residues well exposed to the solvent. Mutagenesis assays were used to investigate functions of these aromatic residues (W476A, Y484A, Y490A). The W476A and Y484A mutations, which are in the grove between the catalytic and CBM46 domains, have larger impact on the enzymatic activity than the Y490A mutation which is outside of the grove. To confirm the importance of tryptophan residues in the binding of polysaccharide substrate, the CelB mutants (H103Q, W156A, W229A, W476A) were used to perform the affinity gel electrophoresis assay. The results clearly indicate that the affinity of W476A and W229A mutants to HEC is much lower than the native protein. As a result, these two mutants migrate much faster in the affinity gel, which indicates W229A and W476A play important roles in substrate binding. Likewise, the W156A mutant also migrates faster than the native protein, but to a much less extent. In contrast, the H103Q mutation does not change the migration speed of CelB (Fig 6). To investigate the importance of CBM_X domain, several aromatic residues in this domain were mutated. The subsequent enzymatic assay indicates that H349A, H366A and W425A have some impact on the enzyme activities. His349 and His366 are exposed on the surface of the protein and they may be involved in the binding of the substrate. Although they are located far from catalytic residues, the polymer nature of the substrate CMC makes it possible to interact with the residues distal from the active site. The reason why W425A mutation is detrimental is unclear.

### Molecular Dynamics Simulation of CelB

**Fig 7:**
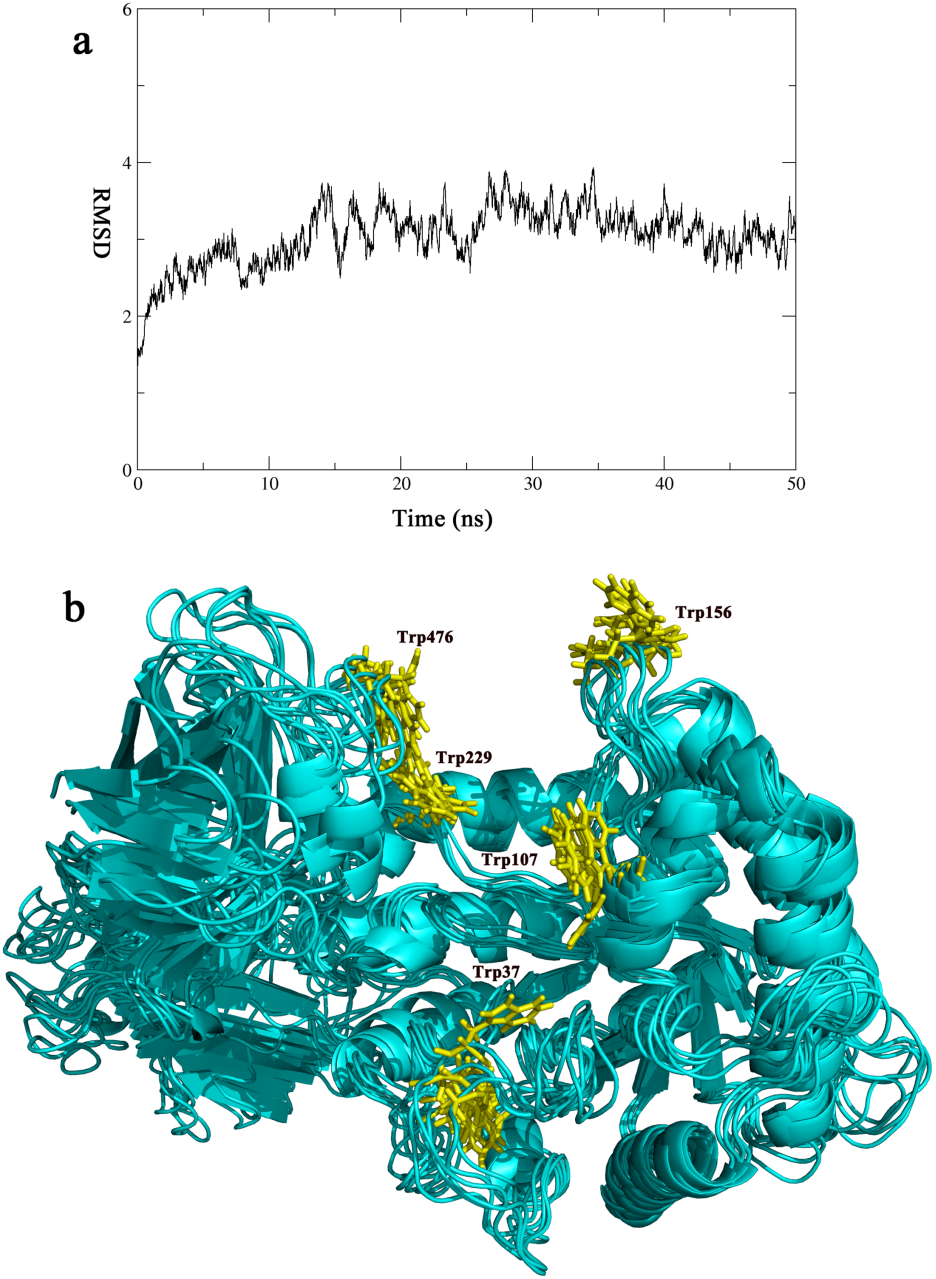
The molecular dynamics simulation of CelB. The CelB structure is simulated over a period of 50 ns. (a) The RMSD Plot. The RMSD of the simulation was plotted against time, according to the simulation trajectories. (b) The cluster representation of CelB structure. The snapshots of the trajectory were taken every 10 ns along the simulation process. The snapshots were overlapped with each other. Only small fluctuation is found and they are mainly concentrated in the loop areas. The tryptophan residues (Trp37, Trp107, Trp156, Trp229, Trp476) are colored in yellow and shown in sticks.

In the classic cellulase system, the CBM domain is connected the catalytic domain by a flexible linker. The CBM domain binds to the cellulosic substrate first. Then the catalytic domain adjusts its position with the flexible linker and find the best orientation on the substrate. In the case of CelB, the CBM46 and catalytic domains are not only bridged by a rigid CBM_X domain but also contact each other directly through hydrogen bonds. To investigate the flexibility of the CelB, we performed molecular dynamics (MD) simulation. The result indicates that the overall folding of CelB is very stable throughout the simulation, indicating the CelB is unlikely to bind the substrate through the classic model (Fig 7). However, we notice that the aromatic residues, which are proven to be involved in the substrate binding, are mostly located in the loop regions. These regions undergo structural change during the simulation, which may provide the flexibility required for substrate binding and subsequent product release (Fig 7).

## Discussion

In many cellulases, extended linker sequences are found between the catalytic domains and the CBMs[40, 51]. It was suggested that the linkers help to recruit catalytic domains to the cellulosic substrates after the CBMs bind to the substrates. The presence of CBMs and the unstructured linker sequences help to anchor the catalytic domains on the substrates, therefore, boosts the catalytic rates[52]. The mechanism of the linkers implies that the linker sequences are most useful when the cellulases are used to digest insoluble substrate such as microcrystalline cellulose[53]. The rigid nature of the insoluble substrates requires the cellulases to stay flexible in order to fit the surfaces of the substrates. However, for CelB, all its substrates are soluble[10]. The soluble substrates have the abilities to adjust their positions in solution so that they can fit in the active site of CelB. As indicated by the MD simulation, the overall structure of CelB stays unchanged over the 50ns simulation process. The rigid nature of CelB indicates that it uses a different mechanism to anchor the enzyme to the substrate molecule. In order to catch the substrate with higher efficiency, CelB expands its substrate-binding site beyond the catalytic domain. The binding cleft spanning two domains provides much bigger binding surface and more aromatic residues for the substrate to recognize. This mechanism helps CelB to catch the soluble substrate even if it does not have the flexible linkers.

The CelB structure represents a rare example in cellulases that the catalytic domain, a CBM_X domain and a CBM46 domain form a tightly-packed L-shaped structure. Although rare in cellulases, such tight arrangement could be found in other GHs such as dextrinase and cyclodextrin glycosyltransferase, which are both starch-converting enzymes[54, 55]. Like the structure of CelB, the CBMs in these GHs are arranged around the catalytic domain. It is possible that the putative CBM(CBM_X) serves both as a spacer as well as the substrate-binding site. As a result, the CBM at the C-terminus is positioned at the opening of catalytic domain and forms part of the long substrate-binding cleft. CelB and the starch-converting enzymes may share the similar mechanism as far as substrate recruitment.

The CelB is a halophilic cellulase. Its activities increase 10 fold in the presence of 2.5M NaCl[10]. Such property has been observed with other endoglucanases, but to a much less extent[56, 57]. No significant structure change was observed when salt concentration was increased, which rules out the possibility of refolding of the protein under high salt[10]. The surface of CelB is highly negatively charged (Fig 5). However, there are several small positive charged patches present. It is possible that under low salt conditions, these positively charged patches serve as an attaching point for the neighboring highly negatively charged CelB molecules. And these molecules may form oligomer through electrostatic interactions. In fact, as observed by dynamic light scattering, the diameters of particles in CelB solution increase significantly under the low salt conditions (data not shown). The formation of such oligomers would prevent the substrate binding and, therefore, hinder the catalytic activities.

## Supporting Information

S1 Fig The sequence alignment of several cellulases with a GH5 catalytic domain and a CBM46 domain. (DOCX)

S2 Fig The limited proteolysis of CelB mutants. (DOCX)

S1 Table Primers for *celB* gene amplification and site-directed mutagenesis. (DOCX)
