# Supplementary material for: Structural Insight of a Trimodular Halophilic Cellulase with a Family 46 Carbohydrate-Binding Module
Source: PLoS One. 2015 Nov 12;10(11):e0142107. doi: 10.1371/journal.pone.0142107 (PMC4643050; doi:10.1371/journal.pone.0142107)
Supplement: S1 Fig — (DOCX) [file pone.0142107.s001.docx]

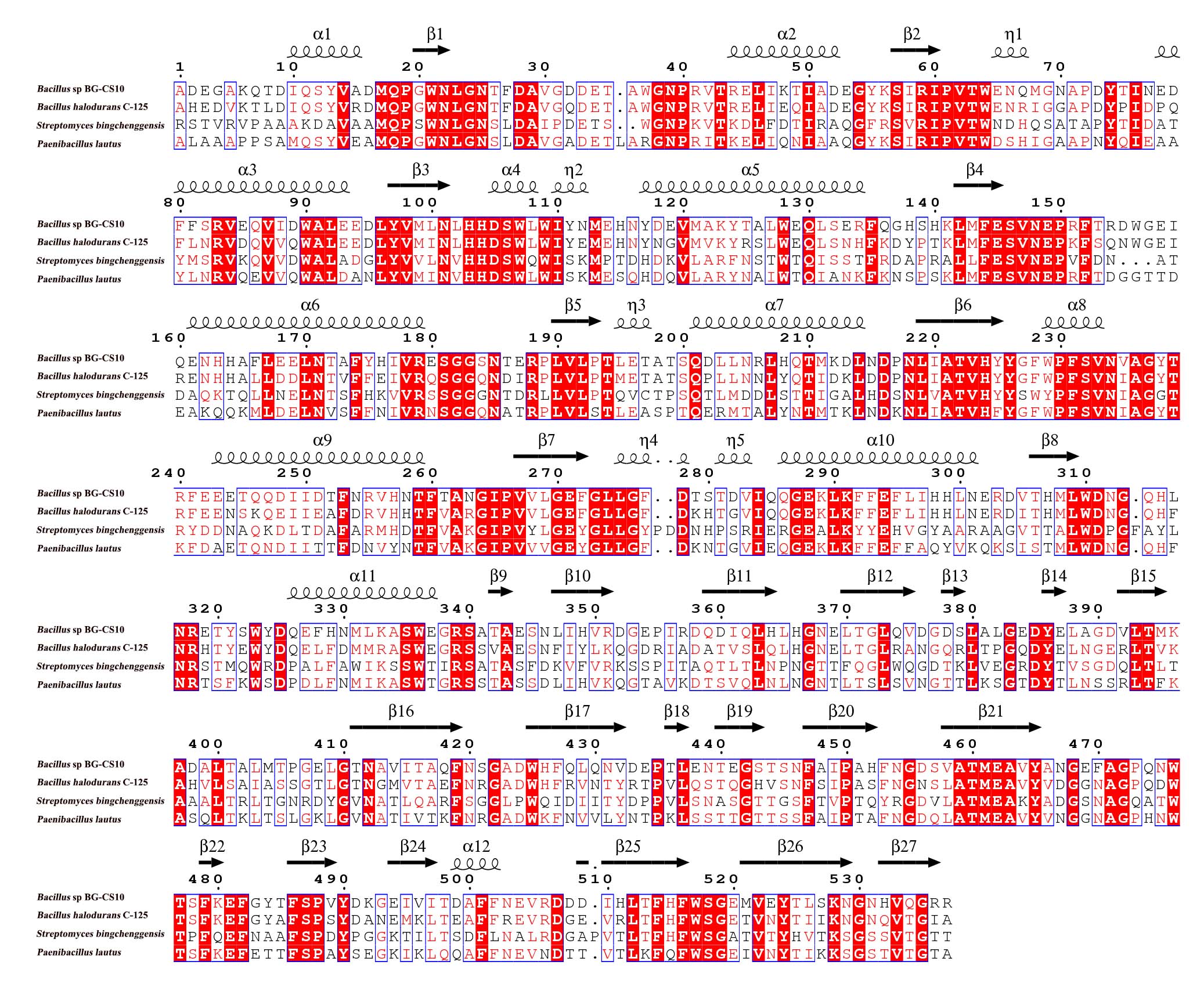


S1 Fig. **The sequence alignment of several cellulases with a GH5 catalytic domain and a CBM46 domain.** The sequences of cellulases from *Bacillus* sp. BG-CS10, *Bacillus halodurans* C-125, *Streptomyces bingchenggensis*, *Paenibacillus lautus* are aligned with each other. The alignment is annotated with the secondary structure elements in CelB. The α helices are designated as α; the β strands are designated as β; the 3/10 helices are labeled as η.
