# Supplementary material for: Structural Insight of a Trimodular Halophilic Cellulase with a Family 46 Carbohydrate-Binding Module
Source: PLoS One. 2015 Nov 12;10(11):e0142107. doi: 10.1371/journal.pone.0142107 (PMC4643050; doi:10.1371/journal.pone.0142107)
Supplement: S2 Fig — (DOCX) [file pone.0142107.s002.docx]

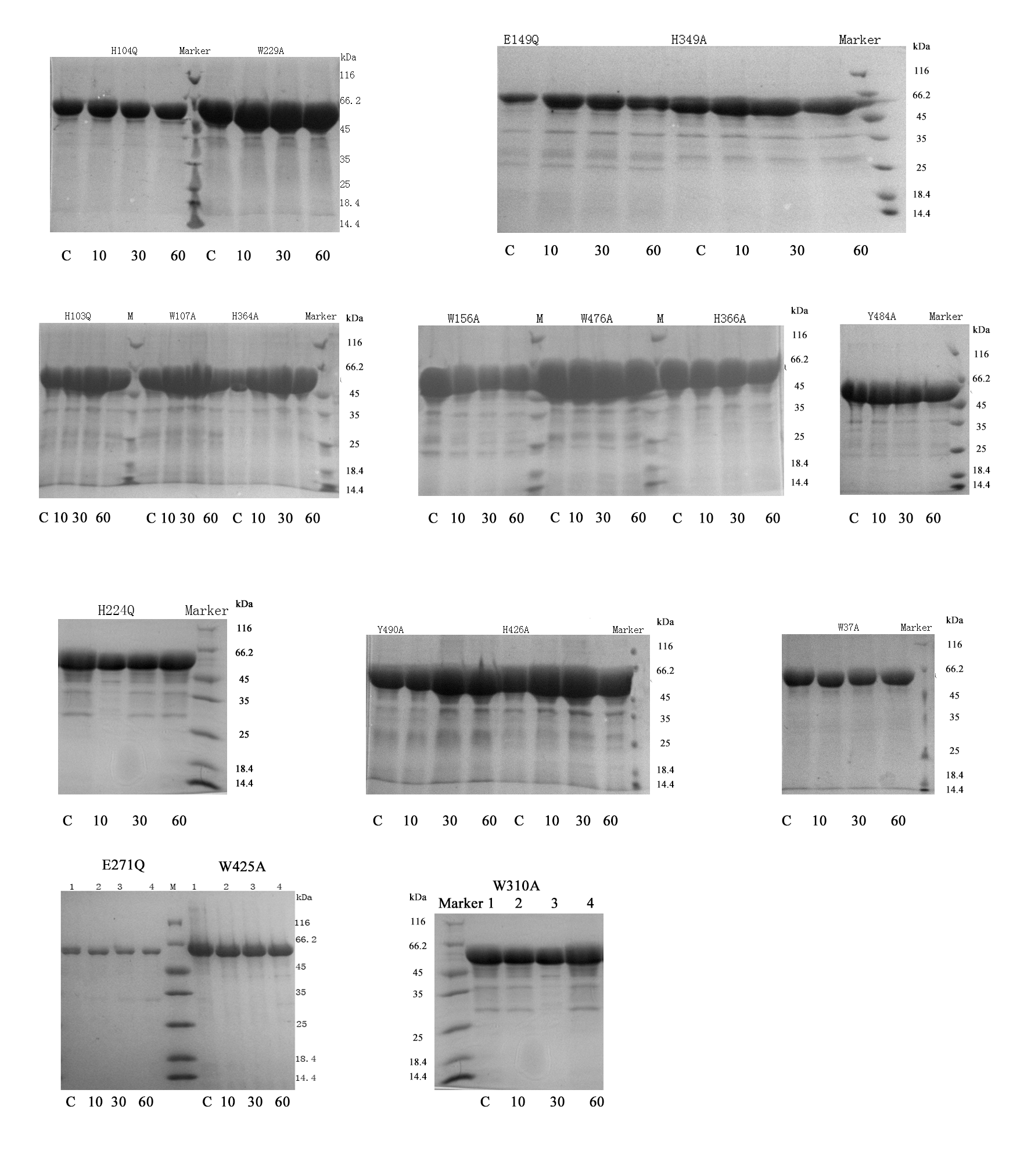


S2 Fig. **The limited proteolysis of CelB mutants.** The CelB mutants were digest with Trypsin for 10 min, 30 min, 60 min before they were revolved on a SDS PAGE gel. The control lane is marked as C, and the lanes for the 10 min, 30 min and 60 min digestion are marked as 10, 30 and 60 respectively. The results show that the limited proteolysis does not affect the migration of the mutant protein even after 60-min incubation. This is a good indication that these proteins are well folded.
