# Supplementary material for: Structural Insight of a Trimodular Halophilic Cellulase with a Family 46 Carbohydrate-Binding Module
Source: PLoS One. 2015 Nov 12;10(11):e0142107. doi: 10.1371/journal.pone.0142107 (PMC4643050; doi:10.1371/journal.pone.0142107)
Supplement: S1 Table — (DOCX) [file pone.0142107.s003.docx]

**S1 Table** Primers for *celB* gene amplification and site-directed mutagenesis.

| Name of primers | Sequence (5’-3’) | Name of primers | Sequence (5’-3’) |
| --- | --- | --- | --- |
| W37A_F | CACACGAGGATTCCCGGCAGCTGTTTCATCATC | W37A_R | GATGATGAAACAGCTGCCGGGAATCCTCGTGTG |
| H103Q_F | CAGCCATGAATCATGCTGCAGATTTAACATGAC | H103Q_R | GTCATGTTAAATCTGCAGCATGATTCATGGCTG |
| H104Q_F | ATATGTCATGTTAAATCTGCATCAAGATTCATGGCTGTGGATATATA | H104Q_R | TATATATCCACAGCCATGAATCTTGATGCAGATTTAACATGACAT |
| W107A_F | ATTGTGTTCCATATTATATATCCACAGGGCTGAATCATGATGCAGATTTAACATGAC | W107A_R | GTCATGTTAAATCTGCATCATGATTCAGCCCTGTGGATATATAATATGGAACACAAT |
| E149Q_F | CATAAATTAATGTTTGAGAGTGTCAATCAGCCTCGGTTTACG | E149Q_R | CGTAAACCGAGGCTGATTGACACTCTCAAACATTAATTTATG |
| W156A_F | CTTGAATCTCTCCGGCATCTCGCGTAAACCG | W156A_R | CGGTTTACGCGAGATGCCGGAGAGATTCAAG |
| H224Q_F | CCAGAAGCCATAATACTGAACTGTGGCTATCAAG | H224Q_R | CTTGATAGCCACAGTTCAGTATTATGGCTTCTGG |
| W229A_F | CACAGTTCATTATTATGGCTTCGCGCCATTTAGTGTAAATGTAGCAG | W229A_R | CTGCTACATTTACACTAAATGGCGCGAAGCCATAATAATGAACTGTG |
| E271Q_F | TCCTGTCGTATTGGGTCAATTTGGCTTGTTAGGCT | E271Q_R | AGCCTAACAAGCCAAATTGACCCAATACGACAGGA |
| H349A_F | GCTACAGCAGAGTCTAATTTAATTGCTGTGAGGGACGGGG | H349A_R | CCCCGTCCCTCACAGCAATTAAATTAGACTCTGCTGTAGC |
| H364A_F | TAGAGACCAAGATATACAGCTTGCTTTACACGGAAATGAGCTAACAG | H364A_R | CTGTTAGCTCATTTCCGTGTAAAGCAAGCTGTATATCTTGGTCTCTA |
| H366A_F | CCAATTAGAGACCAAGATATACAGCTTCATTTAGCCGGAAATGAGCTAACAG | H366A_R | CTGTTAGCTCATTTCCGGCTAAATGAAGCTGTATATCTTGGTCTCTAATTGG |
| W425A_F | CTCAATTTAATTCTGGAGCTGACGCGCATTTTCAACTACAGAATGTAG | W425A_R | CTACATTCTGTAGTTGAAAATGCGCGTCAGCTCCAGAATTAAATTGAG |
| H426A_F | ATTTAATTCTGGAGCTGACTGGGCTTTTCAACTACAGAATGTAGACG | H426A_R | CGTCTACATTCTGTAGTTGAAAAGCCCAGTCAGCTCCAGAATTAAAT |
| W476A_F | CCGAATTCTTTAAACGATGTGGCATTTTGTGGTCCGGCAAATTC | W476A_R | GAATTGCCGGACCACAAAATGCCACATCGTTTAAAGAATTCGG |
| Y484A_F | CATCGTTTAAAGAATTCGGTGCTACCTTTTCACCTGTTTACG | Y484A_R | CGTAAACAGGTGAAAAGGTAGCACCGAATTCTTTAAACGATG |
| Y490A_F | GTTATACCTTTTCACCTGTTGCCGACAGGGGAGAAATTGTC | Y490A_R | GACAATTTCTCCCCTGTCGGCAACAGGTGAAAAGGTATAAC |
| CelB_F | CGCGGATCCGATGAAGGTGCTAAGCAAACAGATATTC | CelB_R | AGCGTCGACTTATCGTCTACCTTGAACATGGTTTCC |
